# Reversed Apico-Basal Myocardial Relaxation Sequence During Exercise in Long QT Syndrome Mutations Carriers With History of Previous Cardiac Events

**DOI:** 10.3389/fphys.2021.780448

**Published:** 2022-02-07

**Authors:** Dafni Charisopoulou, George Koulaouzidis, Annika Rydberg, Michael Y. Henein

**Affiliations:** ^1^Institute of Public Health and Clinical Medicine, Umea University, Umeå, Sweden; ^2^Division of Pediatric Cardiology, Department of Pediatrics, Amalia Children’s Hospital, Radboud University Medical Center, Nijmegen, Netherlands; ^3^Academic Centre for Congenital Heart Disease, Nijmegen, Netherlands; ^4^Department of Biochemical Sciences, Pomeranian Medical University, Szczecin, Poland; ^5^Department of Clinical Sciences, Paediatrics, Umea University, Umeå, Sweden

**Keywords:** arrhythmia, long QT syndrome, exercise stress echocardiogram, speckle-tracking echocardiography, diastolic function, myocardial relaxation sequence

## Abstract

**Background:**

Recent echocardiography studies in inherited long QT syndrome (LQTS) have shown left ventricular (LV) myocardial relaxation disturbances to follow markedly prolonged and dispersed mechanical contraction.

**Aim:**

We used speckle-tracking echocardiography to assess disturbances in LV myocardial relaxation sequence during exercise and their relationship to symptoms.

**Methods:**

Forty seven LQTS patients (45 ± 15 years, 25 female and 20 symptomatic, LVEF: 65 ± 6%) and 35 controls underwent exercise echocardiogram using Bruce protocol. ECG and echo parameters were recorded at rest, peak exercise (p.e.) and recovery.

**Results:**

Between patients and controls there were no differences in age, gender, HR or LVEF. At p.e, patients had longer time to LV longitudinal E_SR_ (tE_SR_) at all three LV segments; basal (*p* < 0.0001), mid- cavity (*p* = 0.03) and apical (*p* = 0.03) whereas at rest such difference was noted only at base (*p* = 0.0007). Patients showed reversed apico-basal relaxation sequence (ΔtE_SRbase–apex_) with early relaxation onset occurring later at base than at apex, both at rest (49 ± 43 vs. –29 ± 19 ms, *p* < 0.0001) and at p.e. (46 ± 38 vs. –40 ± 22 ms, *p* < 0.0001), particularly in symptomatic patients (69 ± 44 vs. 32 ± 26, *p* < 0.0007). ΔtE_SRbase–apex_ correlated with longer QTc interval, lower E_SR_ and attenuated LV stroke volume.

**Conclusion:**

LQTS patients show reversed longitudinal relaxation sequence, which worsens with exercise, particularly in those with previous cardiac events.

## Introduction

Novel cardiovascular imaging techniques have recently offered new insights into the behavior of the left ventricular (LV) myocardium in inherited long QT syndrome (LQTS). This is no longer regarded as a single electrical phenomenon caused by mutations in genes encoding for cardiac ion channels (Schwartz and Ackerman, 2013). Myocardial function analysis, using speckle-tracking echocardiography in LQTS patients, has shown prolonged action potential and repolarization dispersion to be related to prolonged and markedly dispersed myocardial contraction (Haugaa et al., 2010; Charisopoulou et al., 2020). Such electromechanical abnormalities are more pronounced in mutation carriers with history of adverse cardiac events (Haugaa et al., 2009; Leren et al., 2015; Charisopoulou et al., 2018b).

The resting mechanical contraction disturbances, in LQTS patients, have been shown, in a recent speckle-tracking echocardiography study, to deteriorate further with exercise and to result in myocardial relaxation disturbances in the form of delayed onset of relaxation, shortened LV filling time with its implication on inadequate LV stroke volume (SV) response (Charisopoulou et al., 2018a). Brado et al. (2017) employed phase-contrast magnetic resonance imaging in a small group of LQTS pediatric patients, at rest, and suggested additional evidence for reversed apico-basal myocardial relaxation sequence in longitudinal LV function.

The aim of this study was to assess disturbances in LV apico-basal myocardial relaxation sequence during exercise and their relationship to symptoms in LQTS mutation carriers.

## Materials and Methods

### Study Population

We enrolled 47 patients with genetically confirmed LQTS (36 LQTS1 and 11 LQTS2), who were under regular follow-up at Umea University Hospital (Table 1). They were compared with 37 healthy subjects (control group), matched for age (45 ± 15 vs. 47 ± 13 years, *p* = 0.2) and gender (53 vs. 54% females, *p* = 0.3). None of the participants had electrocardiographic (ECG) or echocardiographic changes suggestive of underlying ischemic heart disease. There was also no evidence for bundle branch block (BBB) on the recorded 12 lead ECGs of the participants. No patient had evidence for other cardiac or valve disease. All patients underwent exercise echocardiographic examination with the following set target: exhaustion, symptoms, arrhythmia or significant drop in systolic blood pressure (> 20 mmHg).

**TABLE 1:** Characteristics of LQTS patients and controls.

	LQTS *n* = 47	Control *n* = 35	*p*-value
Age (years)	45 ± 15	47 ± 13	0.2
Gender, female (n,%)	25 (53%)	19 (54%)	0.3
Genotype (*n*,%)			
LQTS type 1	36 (77%)	n/a	
LQTS type 2	11 (33%)		
Symptomatic (*n*,%)	20 (43%)	0	
B-blocker therapy (*n*,%)	25 (53%)	0	
Symptomatic	14		
Asymptomatic	11		
Maximal workload (Watts)	125 ± 26	131 ± 35	0.3

*Symptomatic, documented history of previous cardiac events.*

The LQTS patients were classified into symptomatic or asymptomatic based on previously documented cardiac events such as cardiac arrest, syncope or ventricular arrhythmias.

### Exercise Echocardiography Protocol

The exercise test was performed using a supine bicycle, General Electric- GE ergometer (model 900, Ergoline GmbH, Bitz, Germany). Patients and controls exercised in a semi-supine position (slightly left lateral tilt) and in stages of incremental 10 Watt increase of workload, every 2 min. Echocardiographic recordings, ECG and blood pressure (BP) were made at three stages: (i) at rest (just before exercise), (ii) at peak exercise (p.e.), achieving 85% of the maximum predicted heart rate (HR) for age, and (iii) at 4 min into recovery.

Throughout the exercise test, a 12-lead ECG was continuously recorded, at 25 mm/s with a conventional electrocardiograph, with built-in measurements software. Manual measurements of RR, QT and QRS were performed at the end of each phase. Only resting measurements were available digitally from the electrocardiograph, exercise measurements were made manually throughout all stages. The QT interval was measured from the onset of the QRS complex to the end of the T-wave at the point of the T wave descending limb intersection with the isoelectric line. QT values were corrected for HR, using the Bazett formula (QT_c_ = QT/√RR). We calculated QTc percentage changes (ΔQTc,%) from baseline to p.e. and recovery.

The echocardiograms were recorded using a Vivid 7 echocardiograph (GE, Horten, Norway) equipped with an adult 1.5–4.3 MHz phased-array transducer. Digital loops of three cardiac cycles were acquired at the end of each exercise phase from the standard apical four-, two-chamber views and parasternal long-axis views. All recordings were made with a superimposed ECG (Lead II).

### Echocardiogram Analysis

The echocardiogram analysis was performed blindly and off-line on a dedicated work station (EchoPAC, version 110.1.0). LV ejection fraction was estimated using the Simpson’s biplane method and stroke volume (SV) as the product of the LV outflow tract velocity time integral (VTI) and the cross-sectional area. LV Doppler filling velocities (E and A waves) were recorded from the apical four chamber view with the sample volume placed at the orifice of the mitral valve. The recommendations of the European and American society of Echocardiography were used to assess LV diastolic function and LV filling pressures (Nagueh et al., 2008).

**FIGURE 1:**
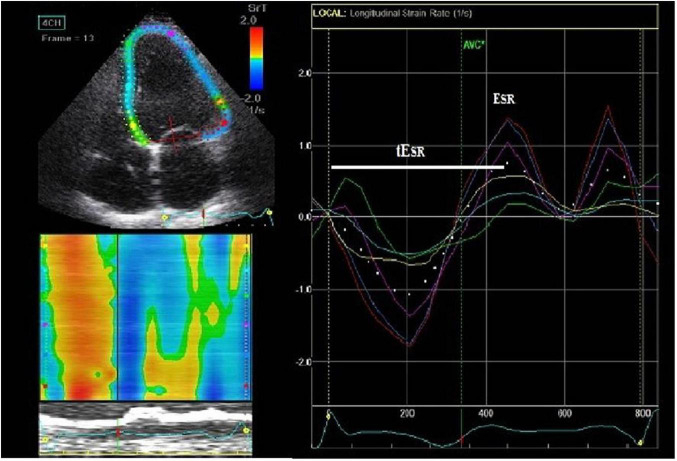
Speckle-tracking 2D-strain rate imaging (from an apical 4-chamber view) with interposed ECG trace. On the end-diastolic frame, we manually traced the left ventricular (LV) endocardial border (left upper figure). The epicardial contour is automatically drawn by the software. Then it is adjusted manually so that the LV wall thickness fits the region of interest (ROI). ROIs are subsequently divided into the standard segments (left upper figure) and regional longitudinal strain rate (SR) curves are produced (right figure). Subsequently, LV basal, middle and apical strain rate parameters were derived. Time to V longitudinal early diastolic SR (tE_SR_), a measure of contraction duration and delay in relaxation, was defined as the time interval from the QRS onset to the peak positive early diastolic SR (E_SR_) early diastolic strain rate.

LV longitudinal early diastolic strain (S) and strain rate (SR) assessment by speckle-tracking echocardiography (STE) were performed at basal, mid, and apical levels (16 LV segments) from the parasternal long axis, apical four- and two-chamber views, at a frame rate of 55–80 frame/s. After selection of the optimal left heart image, the end-diastolic LV endocardial border was manually traced while the epicardial contour was automatically drawn by the software (Figure 1). The region of interest (ROI) was also automatically divided into 6 standard segments for the LV and three slices (basal, mid-cavity, and apical) for the LV free wall and the interventricular septum. The software generated the mean S and SR curves for each ROI. Only subjects in whom all 16 segments could be assessed were included in the study. This was not feasible in two control subjects, due to high body mass index, thus the final study population was made of 47 LQTS patients and 35 controls. LV global early diastolic strain rate (E_SR_) was determined by the average value of the 16 LV segments’ longitudinal peak positive early diastolic SR.

**FIGURE 2:**
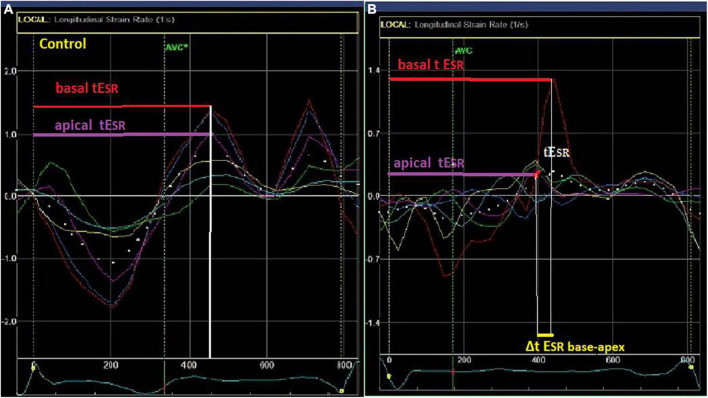
Left ventricular (LV) myocardial strain rate (lower left and right) curves in a control **(A)** and a LQTS patient **(B)** patient. Longitudinal apico-basal myocardial relaxation sequence (ΔtE_SR base–apex_) was defined as the time difference between basal and apical tE_SR._ In the LQTS patient there is delayed early relaxation phase at the base (longer tE_SR_-red arrow) in relation to the apex (purple arrow) with Δt E_SRbase–apex_ > 0.

LV segmental myocardial function was studied using the following parameters: (1) delayed LV longitudinal early diastolic SR (tE_SR_), a measure of contraction duration and delay in relaxation, defined as the time interval from the QRS onset to the peak positive early diastolic SR (Figure 1). The tE_SR_ interval was measured from each of the 16 segmental S and SR curves and the average measurement was calculated for the global LV and the individual, basal, mid-cavity and apical LV segments. All time intervals were given as proportion of the R-R interval (%). (2) The longitudinal apico-basal myocardial relaxation sequence (ΔtE_SR base–apex_) was defined as the time difference between basal tE_SR_ and apical tE_SR_ (Figure 2). (3) The myocardial relaxation sequence was characterized as reversed apico-basal with values ΔtE_SR base–apex_ > 0. With ΔtE_SR base–apex_ values ≤ 0, the relaxation sequence was described as baso-apical (Figure 2).

### Reproducibility of Measurements

In order to assess the inter-observer variability of the speckle-tracking parameters’ measurements, two independent reviewers analyzed the raw images for 20 randomly selected participants. Similarly, the intra-observer variability was assessed based on the analysis of raw data in two different loops. For the QTc, tE_SR_, ΔtE_SR base–apex_ and SV parameters, there was good inter-observer (0.93, 0.984, 0.982, and 0.96, respectively) and intra-observer agreement (0.95 0.987, 0.985, and 0.95, respectively) at all three exercise phases.

### Statistical Analysis

We used Statistical Package for the Social Sciences (SPSS) for Windows (version 13.0, SPSS Inc., Chicago, IL, United States). A two-tailed *P*-value < 0.05 was accepted as a significant difference. Continuous variables are expressed as mean ± standard deviation (SD) and categorical variables as absolute number and percentage (%). We used the Chi-square test to assess if distributions of categorical variables were different from one another. For group comparisons, the Student’s *t-*test was used for variables with normal distribution and the Mann-Whitney *U-*test for non-normally distributed variables. Pearson’s test was used to assess correlations. Multiple regression was performed to calculate odds ratios (ORs) and 95% confidence intervals (95% CIs) for the outcome “previous cardiac events.” The sensitivity and specificity of ΔtE_SRbase–apex_ for identifying previous cardiac events in LQTS patients were investigated using the Receiver Operating Characteristic (ROC) analysis.

### Study Ethics

The study protocol followed the ethical guidelines of the 1975 Declaration of Helsinki and has been approved by the Regional Ethical Review Board (Umeå University, Sweden); Dnr 2011-339-31M. All participants gave informed consent.

## Results

Based on the documented history of previous cardiac events (syncope, cardiac arrest, ventricular tachyarrhythmias), 20 (43%) LQTS patients were classified as symptomatic; three had implantable cardioverter-defibrillator (ICD) inserted. Fourteen (70%) of the symptomatic (previous cardiac events) and 11 (41%) of the asymptomatic LQTS patients were on regular B-blocker therapy. The fourteen treated symptomatic patients experienced cardiac events prior to the initiation of B-blocker therapy whereas none of them were symptomatic while on B-blocker therapy.

### Hemodynamic Parameters, Symptoms During the Exercise Stress

LQTS patients and controls had no significant differences, at rest, p.e. and recovery, in the maximum achieved workload (125 ± 26 vs. 131 ± 35 Watts, *p* = 0.3), in HR (rest: 69 ± 10 vs. 68 ± 10 bpm, *p* = 0.8; p.e.: 121 ± 17 vs. 120 ± 15 bpm, *p* = 0.7 and recovery: 69 ± 10 vs. 68 ± 9 bpm, *p* = 0.7) or in the systolic BP (rest: 117 ± 12 vs. 119 ± 13 mmHg, *p* = 0.8; p.e.: 176 ± 18 vs. 174 ± 14 mmHg, *p* = 0.5 and recovery: 115 ± 15 vs. 118 ± 13 mmHg, *p* = 0.5). Two symptomatic patients developed ventricular bigeminy, close to reaching 85% of maximum predicted HR, with exercise. The rest of the participants remained asymptomatic and did not develop any rhythm disturbances.

### Electromechanical Response to Exercise

#### QTc

LQTS patients had longer QTc than controls at all the three exercise phases (Table 2). The QTc prolonged further at p.e. in patients but shortened in controls (ΔQTc: + 10 ± 9 vs. –5.5 ± 3.8%, *p* < 0.0001).

**TABLE 2:** Left ventricular global and segmental diastolic function at rest, peak exercise and 4 min into recovery.

	LQTS	Control	*p*-value
**REST**			
QTc, *ms*	453 ± 42	413 ± 17	<0.0001
E_SR_, *s^–1^*	1 ± 0.3	2 ± 0.3	<0.0001
E/A	1.1 ± 0.13	1.2 ± 0.35	0.11
E DT, *ms*	220 ± 58	169 ± 18	<0.0001
E/E’ _lateral_	9.2 ± 4.6	6 ± 1.2	0.0002
SV, *ml*	70 ± 6	69 ± 9	0.5
Δ t E_SR_ base-apex, *ms*	49 ± 43	−29 ± 19	<0.0001
t E_SR_,*ms*			
Base	490 ± 59	486 ± 36	0.6
Mid	488 ± 45	498 ± 39	0.3
Apex	467 ± 52	512 ± 44	<0.0001
Cor t E_SR_,*%*			
Base	64 ± 10	56 ± 12	0.0007
Mid	63 ± 9	58 ± 13	0.03
Apex	60 ± 8	59 ± 12	0.6
Peak exercise			
QTc, *ms*	499 ± 45	390 ± 19	<0.0001
E_SR_, *s*^–^*^1^*	1.1 ± 0.4	2.8 ± 0.4	<0.0001
E/A	0.94 ± 0.2	1.2 ± 0.1	<0.03
E DT, *ms*	138 ± 42	101 ± 18	<0.0001
E/E’ _lateral_	12.6 ± 5	6.3 ± 1.7	0.0026
SV, *ml*	68 ± 10	96 ± 11	<0.0001
Δ t E_SR_ base-apex, *ms*	46 ± 38	−40 ± 22	<0.0001
t E_SR_, *ms*			
Base	394 ± 67	316 ± 30	<0.0001
Mid	363 ± 64	340 ± 32	0.03
Apex	348 ± 60	368 ± 28	0.03
Cor t E_SR_,%			
Base	74 ± 10	55 ± 8	<0.0001
Mid	69 ± 9	62 ± 8	0.0001
Apex	66 ± 10	62 ± 9	0.04
Recovery			
QTc, *ms*	479 ± 35	414 ± 20	<0.0001
E_SR_, *s*^–^*^1^*	0.95 ± 0.3	1.9 ± 0.5	<0.0001
E/A	1.12 ± 0.16	1.19 ± 0.24	0.15
E DT, *ms*	227 ± 81	170 ± 16	<0.0001
E/E′ _lateral_	9.3 ± 4.6	6.4 ± 1.31	<0.0001
SV, *ml*	68 ± 8	71 ± 11	0.1
Δ t E_SR_ base-apex, *ms*	37 ± 36	−38 ± 17	<0.0001
t E_SR_, *ms*			
Base	477 ± 64	391 ± 28	<0.0001
Mid	462 ± 63	407 ± 32	<0.0001
Apex	440 ± 62	429 ± 32	<0.0001
Cor t E_SR_,%			
Base	64 ± 13	53 ± 8	0.01
Mid	62 ± 10	55 ± 9	0.0006
Apex	59 ± 12	58 ± 10	0.6

*QTc, QT corrected; ESR, LV global longitudinal early diastolic strain rate; E_SR_, Early diastolic Strain Rate; E/A, ratio of E wave over A wave; E DT, E-wave deceleration time; E/E**′**, ratio of E wave over E**′**; SV, stroke volume; t ESR, Time to early diastolic SR from R (at base, mid and apex); Corrected t ESR, Time to early diastolic SR from R corrected for R-R interval (at base, mid and apex); Δ t ESR base-apex, time difference between base and apex ESR; > 0, apex diastole starts earlier/ < 0, Base diastole starts earlier.*

#### Left Ventricular Systolic Function

Between LQTS patients and controls, there were no significant difference in resting LV EF (65 ± 6 vs. 67 ± 7%, *p* = 0.3). At rest and at recovery, SV was similar between the two groups (Table 2), however, at peak exercise, it became significantly lower in patients compared to controls (Table 2). Also, the SV increase, in response to exercise, was significantly attenuated in patients compared to the controls (Δ SV: + 2 ± 1 vs. 32 ± 4%, *p* < 0.0001).

#### Left Ventricular Longitudinal Diastolic Function

LQTS patients had significantly lower longitudinal E_SR_ than controls, at all three exercise phases (Table 2). In response to exercise, E_SR_ increased but to a lesser extent in patients than controls (Δ E_SR_: + 20 ± 32 vs. + 51 ± 35%, *p* = 0.0001). Also, E/e’ and E wave deceleration time (EDT) parameters, indirect reflectors of LV filling pressure, were higher in the LQTS patients at all three exercise phases (Table 2).

Global LV longitudinal tE_SR_ was longer in patients than in controls at all exercise phases (rest: 61 ± 7 vs. 58 ± 4, *p* = 0.03; peak: 70 ± 6 vs. 56 ± 3, *p* ≤ 0.0001; recovery: 65 ± 7 vs. 57 ± 1, *p* < 0.0001). This was further examined at the LV basal, mid-cavity and apical segments, separately. At rest, patients had more prolonged tE_SR_ at the basal and mid LV cavity regions (*p* < 0.03, Table 2) but apical regions tE_SR_ were not different from controls (*p* = 0.6, Table 2). At peak exercise, patients had longer tE_SR_ at all three LV segments; basal, mid-cavity and apical (*p* < 0.04, Table 2). At recovery, the pattern of segmental delay and inter-relations was similar to that at rest, the tE_SR_ was more delayed at basal and mid-cavity segments (*p* < 0.01) but not at the apex (*p* = 0.6) in patients (Table 2).

#### Apico-Basal Relaxation Dispersion and Sequence

**FIGURE 3:**
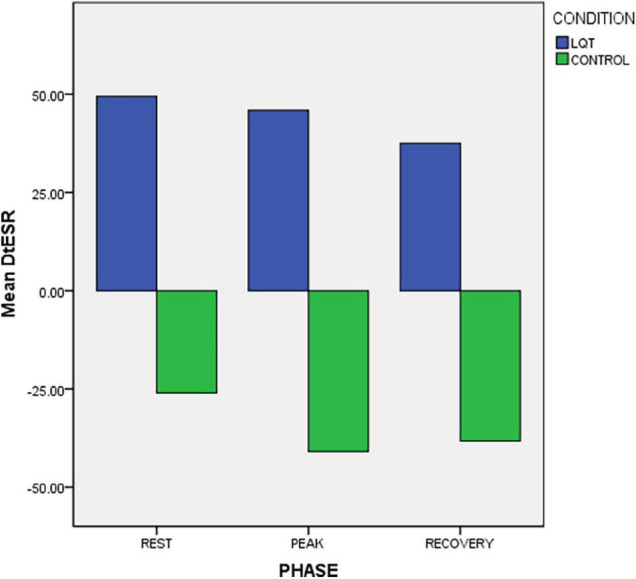
Exercise response of longitudinal apico-basal relaxation sequence (Δ t E_SR_ base-apex) in LQTS mutation carriers and controls. There is reversed relaxation sequence in LQTS with base relaxing later than in apex.

LQTS patients and controls had significantly different longitudinal relaxation pattern which was reflected in the Δt E_SRbase–apex_ (Figure 3). Patients showed delayed early relaxation phase at the base (longer tE_SR_) in relation to the apex (longer tESR), at rest and during exercise, resulting in positive values for Δt E_SRbase–apex_ (Table 2 and Figures 2, 3). This pattern was opposite to controls, in whom tE_SR_ at the apex was longer than at the base, resulting in negative values for Δt E_SRbase–apex_ (Table 2 and Figure 3).

### Electromechanical Correlations

**FIGURE 4:**
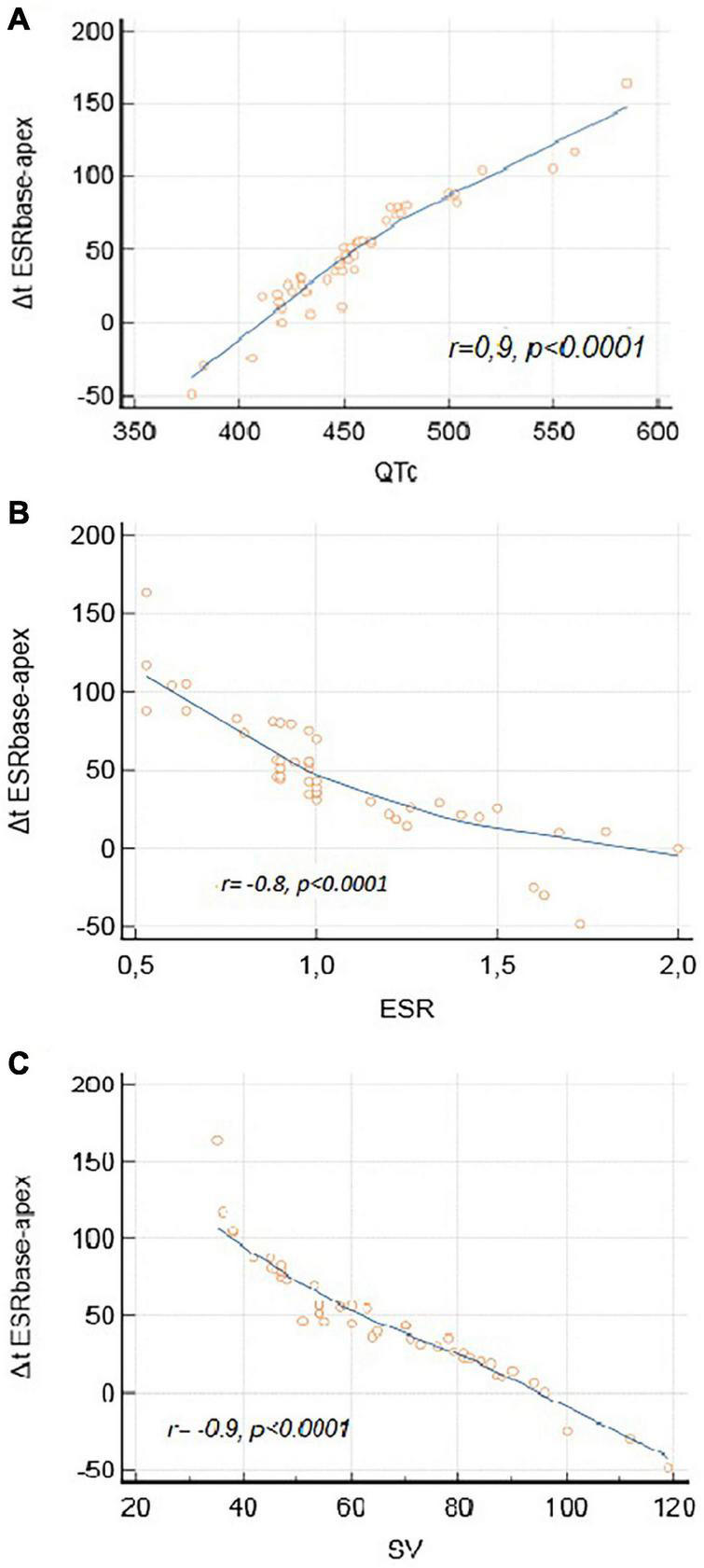
Electromechanical correlations. **(A)** Correlation between Δt E_SRbase–apex_ and QTc. **(B)** Correlation between Δt E_SRbase–apex_ and ESR. **(C)** Correlation between Δt E_SRbase–apex_ and SV.

The magnitude of the apico-basal relaxation dispersion (Figure 4) correlated with longer QTc (*r* = 0.9, *p* < 0.0001), lower E_SR_ (*r* = –0.8, *p* < 0.0001) and attenuated LV stroke volume (*r* = –0.9, *p* < 0.0001).

### Symptomatic vs. Asymptomatic Long QT Syndrome Patients

**FIGURE 5:**
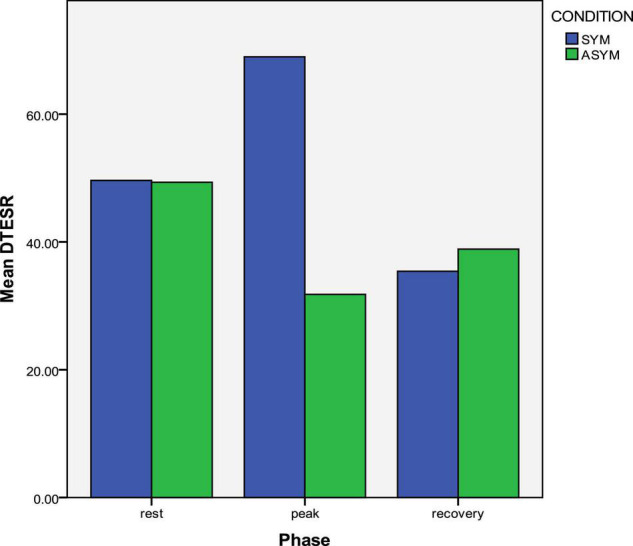
Exercise response of longitudinal apico-basal relaxation sequence in symptomatic and asymptomatic LQTS mutation carriers. The reversed relaxation pattern is more prominent in symptomatic patients at peak exercise.

Symptomatic LQTS patients had significantly lower E_SR_ than asymptomatic patients, at all three exercise phases (Table 3). Reversed LV longitudinal relaxation pattern existed in both symptomatic and asymptomatic patients and it was of similar degree at rest and at recovery, in the two groups (Table 3). However, the reversed relaxation pattern was more pronounced at peak exercise in symptomatic patients in whom the tE_SRbase–apex_ difference between LV base and apex was significantly higher (Table 3 and Figure 5). In a similar pattern, the calculated SV was significantly lower in the symptomatic subgroup at peak exercise (Table 3).

**TABLE 3:** Symptomatic and asymptomatic LQTS mutation carriers.

	Symptomatic	Asymptomatic	*p*-value
**QTc,** *ms*			
Rest	479 ± 43	457 ± 21	0.1
Peak exercise	504 ± 41	479 ± 14	0.003
Recovery	495 ± 39	469 ± 16	0.002
**Global E_SR_**, *s*^–^*^1^*			
Rest	0.92 ± 0.23	1.12 ± 0.3	0.01
Peak exercise	0.92 ± 0.23	1.26 ± 0.36	0.0006
Recovery	0.75 ± 0.19	1.04 ± 0.25	0.0001
**Global t E_SR_**, *ms*			
Rest	64.58 ± 6.9	57.14 ± 3.6	0.0001
Peak exercise	73.32 ± 4.3	67.1 ± 5.6	0.0002
Recovery	69.75 ± 6.3	60.87 ± 60.4	<0.0001
**Δ t E_SR_ base-apex**, *ms*			
Rest	50 ± 35	49 ± 32	0.07
Peak exercise	69 ± 44	32 ± 26	0.0007
Recovery	35 ± 29	39 ± 38	0.7
**SV,** *ml*			
Rest	70 ± 6	69 ± 9	0.5
Peak exercise	68 ± 10	96 ± 11	<0.0001
Recovery	68 ± 8	71 ± 11	0.1

*QTc, QT corrected; ESR, LV global longitudinal early diastolic strain rate; E_SR_, Early diastolic Strain Rate; t ESR, Time to early diastolic SR from R (at base, mid and apex); Corrected t ESR, Time to early diastolic SR from R corrected for R-R interval (at base, mid and apex); Δ t ESR base-apex, time difference between base and apex ESR; > 0, apex diastole starts earlier/ < 0, Base diastole starts earlier; SV, stroke volume.*

On multiple regression analysis which included the parameters of ΔtE_SR base–apex_ and QTc at rest, peak exercise and recovery, only ΔtE_SR base–apex_ at peak exercise (OR 1.4, 95% CI 1.02–1.2; *P* = 0.01) and ΔtE_SR base–apex_ at recovery (OR 1.01, 95% CI 0.92–0.99; *P* = 0.02) proved to be independent predictors of previous cardiac events. ROC analysis showed ΔtE_SR base–apex_ at peak exercise to be the best identifier (AUC 0.8, 95% CI 0.66–0.9, *P* = 0.009) of patients with previous cardiac events followed by ΔtE_SR base–apex_ at recovery (AUC 0.5, 95% CI 0.37–0.6).

### Effect of B-Blocker Therapy

Between treated with B-blockers and untreated patients, there were no significant differences in QTc at rest (452 ± 46 vs. 454 ± 37 ms, *p* = 0.8), peak exercise (491 ± 37 vs. 509 ± 52 ms, *p* = 0.1) or recovery (481 ± 38 vs. 477 ± 33 ms, *p* = 0.7). However, at peak exercise, untreated patients had more pronounced apico-basal relaxation dispersion and reversed sequence (Δt E_SRbase–apex_) than treated patients (17 ± 13 vs. 76 ± 28 ms, *p* < 0.0001) despite no noted differences at rest (55 ± 28 vs. 43 ± 38 ms, *p* = 0.1). In a similar way estimated LV SV was lower at peak exercise in untreated than in treated patients (73 ± 15 vs. 60 ± 17 ml, *p* < 0.01) whereas no differences were noted at rest (75 ± 13 vs. 76 ± 15 ml, *p* = 0.8).

### Exercise Response According to Genotype

At rest, there were no differences between the LQT1 and the LQT2 patients in QTc (458 ± 15 vs. 451 ± 10, *p* = 0.8) or Δt E_SRbase–apex_ (32 ± 42 vs. 45 ± 55, *p* = 0.4). However, at peak exercise, LQT1 patients had longer QTc (503 ± 47 vs. 458 ± 41, *p* = 0.03), greater Δt E_SRbase–apex_ (54.5 ± 34 vs. 19 ± 43, *p* = 0.006) and more attenuated SV response (ΔSV: + 2 ± 0.5 vs. + 4 ± 1%, *p* < 0.0001) than LQT2 patients. At recovery, although there were no significant differences in QTc (477 ± 34 vs. 488 ± 38, *p* = 0.3), LQT2 patients presented greater Δt E_SRbase–apex_ (–26 ± 65 vs. 96 ± 60, *p* < 0.0001) and more attenuated SV response (ΔSV: 3 ± 2 vs. –1 ± 2%, *p* < 0.0001).

## Discussion

### Findings

Our results showed reversed longitudinal relaxation sequence with contraction duration being more prolonged at LV base than at LV apex in LQTS mutation carriers opposite to age and gender matched controls. These disturbances worsened at peak exercise and were more pronounced in patients with history of previous cardiac events and those untreated with B blockers. This apico-basal dispersion in the longitudinal direction was strongly related to longer QTc, impaired LV diastolic function and attenuated stroke volume response to exercise.

### Findings Interpretation

Previous studies (Charisopoulou et al., 2018b,^2020^) in the same cohort of LQTS patients have shown prolonged and markedly dispersed mechanical contraction and negative electromechanical window, more pronouncedly in patients with previous cardiac events. These disturbances increased during exercise and were associated with delayed myocardial relaxation, which in turn related to attenuated stroke volume response to exercise. These associations, along with the fact that organized electromechanical coupling at the onset of diastole is important for adequate left ventricular filling and effective myocardial function (Cordeiro et al., 2004; Sengupta et al., 2006), sparkled our interest to assess further, in the same cohort of LQTS patients, the myocardial behavior during early relaxation and the longitudinal apico-basal relaxation sequence and their potential contribution to the development of symptoms.

Our LQTS patients showed clear evidence for delayed longitudinal myocardial relaxation as a result of prolonged myocardial contraction. This delay appears more pronounced at the LV basal segments compared to the apex. As a result, there was not only an increased apico-basal dispersion in the onset of LV longitudinal relaxation but also a reversed longitudinal relaxation sequence; the onset of basal segment relaxation occurred later than the apex in LQTS patients as opposed to the controls. The magnitude of this apico-basal relaxation dispersion, as reflected by the Δt E_SRbase–apex_, was not an independent mechanical disturbance but strongly correlated with the severity of repolarization disturbances (prolonged QTc). This finding provides additional evidence for the presence of electromechanical (EM) coupling disturbances in LQTS (Odening et al., 2013; Charisopoulou et al., 2018b), irrespective of instigator. Organized EM coupling dispersion at the onset of diastole, has been shown to be essential for efficient myocardial performance; being related to calcium homeostasis heterogeneities which are necessary for adequate myocardial lengthening during diastole (Cordeiro et al., 2004; Sengupta et al., 2006). However, in our LQTS patient group, the EM heterogeneity pattern at the beginning of relaxation appears disorganized leading to an abnormal reversed longitudinal relaxation sequence. Our findings also showed that those EM disturbances at the onset of myocardial relaxation became more pronounced during stress, particularly in patients with history of previous cardiac events. The magnitude of this early relaxation disturbance (Δt E_SRbase–apex_) correlated with decreased early diastolic longitudinal function (E_SR_) and with attenuated LV stroke volume response to exercise.

These findings may indicate an additional predisposing factor for arrhythmias in LQTS patients. Markedly prolonged and dispersed repolarization in LQTS is known to promote re-entry arrhythmias (Antzelevitch, 2008). Such repolarization abnormalities have also been shown to be associated with prolonged mechanical contraction and marked apico-basal and transmural mechanical heterogeneities (Brado et al., 2017; Charisopoulou et al., 2018b) in LQTS mutation carriers. Prolonged myocardial Ca^2^ + overload as a result of prolonged action potential in these patients induces early and late potentials which trigger mechanical post-systolic contractions and thus tachyarrhythmias (Ter Bekke and Volders, 2012). In our LQTS cohort the longitudinal relaxation sequence appeared to be disturbed with a reversed apex to base progression; this was related to impaired LV myocardial relaxation and attenuated stroke volume response during exercise. Myocardial relaxation onset proceeding from base to apex is important in generating negative LV pressure which facilitates adequate early LV filling. It also plays a role in reducing shear forces within the LV cavity during ejection (Ashikaga et al., 2004). So a reversed apico-basal relaxation sequence may result in increased LV diastolic pressure and reduced LV filling, especially during stress, when filling time is also expected to shorten due to increased heart rate (Charisopoulou et al., 2018a). The end result of such changes can be amplified subendocardial flow (Hoffman and Buckberg, 1975; Leren et al., 2015) leading to decreased myocardial reserve with inadequate myocardial blood flow and stroke volume response during stress (Sinha et al., 2021). Such subendocardial ischemia has been previously shown to be accompanied by a series of ionic and biochemical disturbances which can lead to myocardial electrical destabilization and trigger arrythmias (Ghuran and Camm, 2001). These need to be further investigated.

Our results showed also that LQTS patients treated with B-blockers had less pronounced apico-basal relaxation sequence disturbances and less SV attenuation during peak exercise even though there were no differences at rest. This may be related to the ability of B-blockers to reduce the effects of sympathetic overstimulation, thus lowering the heart rate (Medina et al., 2000). B-blockers were shown to reduce the ventricular repolarization dispersion which may trigger arrythmias in LQT1 and LQT2 patients (Singh, 2005). Previous studies have also shown less pronounced contraction duration prolongation in LQT1 and LQT2 patients during stress (Haugaa et al., 2009; Charisopoulou et al., 2020). These may suggest the presence of less electro-mechanical disturbances at peak stress and better control of symptoms with B-blocker therapy.

Finally, in our cohort, LQT1 patients had greater apico-basal relaxation dispersion at peak exercise than LQT2 patients in whom such disturbances were more exaggerated at the recovery phase. These findings may be the result of the different response to adrenergic stimulation in LQT1 and LQT2 mutation carriers (Sy et al., 2010). In LQT1 genetic changes in the slowly activating delayed rectifier potassium current (IKs) are responsible for more pronounced repolarization and EM disturbances during exercise. In contrast, genetic changes in the delayed rectifier potassium current (IKr) in LQT2 patients can relate to the more pronounced disturbances noted during the recovery phase in this group (Cheng and Kodama, 2004). Despite the above differences, further studies with better representation of the two genotype groups are needed to examine variations in the EM response to exercise in LQT1 and LQT2 mutation carriers.

### Clinical Implications

Our study shows the value of exercise stress test and speckle tracking imaging in the assessment of LQTS patients, since identifying those at risk for developing adverse cardiac events remains a challenge. LQTS mutation carriers at rest appeared to have reversed longitudinal relaxation sequence with more prolonged contraction duration at LV base than at LV apex. These EM coupling disturbances worsen at peak exercise and are more pronounced in patients with history of previous cardiac events. These abnormalities are of a lesser degree in patients receiving B-blockers. Thus, measuring EM response to exercise may have an additional value in identifying patients at risk of arrhythmias and in guiding toward optimal management.

### Study Limitations

As a modest number of subjects were included in the study, our results need to be reproduced in a larger cohort of patients with and without symptoms, and with different genotypes. Difficulties in defining the end of T wave may arise especially due to motion artifacts from exercise. Small time differences between groups could be underestimated as a result of the frame rate limitations, particularly for time differences < 20 ms. In order to overcome this and to better describe the longitudinal relaxation sequence, we used the parameter Δ t ESR base-apex; early relaxation starts later at the basal than the apical LV segments if Δ t ESR base-apex > 0 and earlier in the basal than in the apical LV segments if Δ t ESR base-apex < 0. There was no significant difference in the heart rate at peak exercise between controls and patients, but this value was below the age- predicted in both groups. This could be explained by lack of fitness in the control group, and possibly by the effect of B-blockers in the LQTS group (70% of symptomatic and 44% of the asymptomatic patients were on β-blockers). Finally, cardiac events were identified based on the previously documented history, as the LQTS patients were asymptomatic during exercise, except the two patients in whom the exercise test was terminated prematurely due to arrhythmia.

## Conclusion

Although LQTS was initially considered to be the result of sole electrical abnormalities, the use of speckle-tracking echocardiography has shown evidence for associated mechanical contraction and relaxation disturbances. Additionally, our results showed reversed longitudinal relaxation sequence with early relaxation phase starting later at LV base compared to the apex. These disturbances worsened at peak exercise, especially in patients with previous adverse cardiac events, and were related to longer QTc, impaired LV diastolic function and attenuated SV response to exercise.

## Data Availability Statement

The raw data supporting the conclusions of this article will be made available by the authors, without undue reservation.

## Ethics Statement

The studies involving human participants were reviewed and approved by the study protocol followed the ethical guidelines of the 1975 Declaration of Helsinki and has been approved by the Regional Ethical Review Board (Umeå University, Sweden); Dnr 2011-339-31M. All participants gave informed consent. The patients/participants provided their written informed consent to participate in this study.

## Author Contributions

DC designed the protocol of the study, submitted to the ethical committee for approval, collected, analyzed, and interpreted the data, drafted the manuscript, and approved the submitted manuscript. GK contributed to the collection, analysis, and interpretation of the data and approved the submitted manuscript. AR and MH contributed in the conception and design of the study, involved in the interpretation of the data, revised critically the manuscript and approved the submitted manuscript. All authors have seen and agreed with the contents of the manuscript and there is no financial interest to report.

## Conflict of Interest

The authors declare that the research was conducted in the absence of any commercial or financial relationships that could be construed as a potential conflict of interest.

## Publisher’s Note

All claims expressed in this article are solely those of the authors and do not necessarily represent those of their affiliated organizations, or those of the publisher, the editors and the reviewers. Any product that may be evaluated in this article, or claim that may be made by its manufacturer, is not guaranteed or endorsed by the publisher.
